# Editorial: Health services and economic inequalities through the lens of sustainable development

**DOI:** 10.3389/fpubh.2026.1858036

**Published:** 2026-05-12

**Authors:** Vahid Taghvaee, Behnaz Saboori, Faeze Akbariresketi, Moosa Tatar

**Affiliations:** 1 Faculty of Law and Administration, University of Silesia in Katowice, Poland; 2 Department of Natural Resource Economics, Sultan Qaboos University, Muscat, Oman; 3 University of Greifswald, Greifswald, Germany; 4 Department of Pharmaceutical Health Outcomes and Policy, University of Houston, Houston, TX, United States

**Keywords:** economic development, economy, equalities, health, inequality, services, sustainabilify, sustainable development

**Figure 1:**
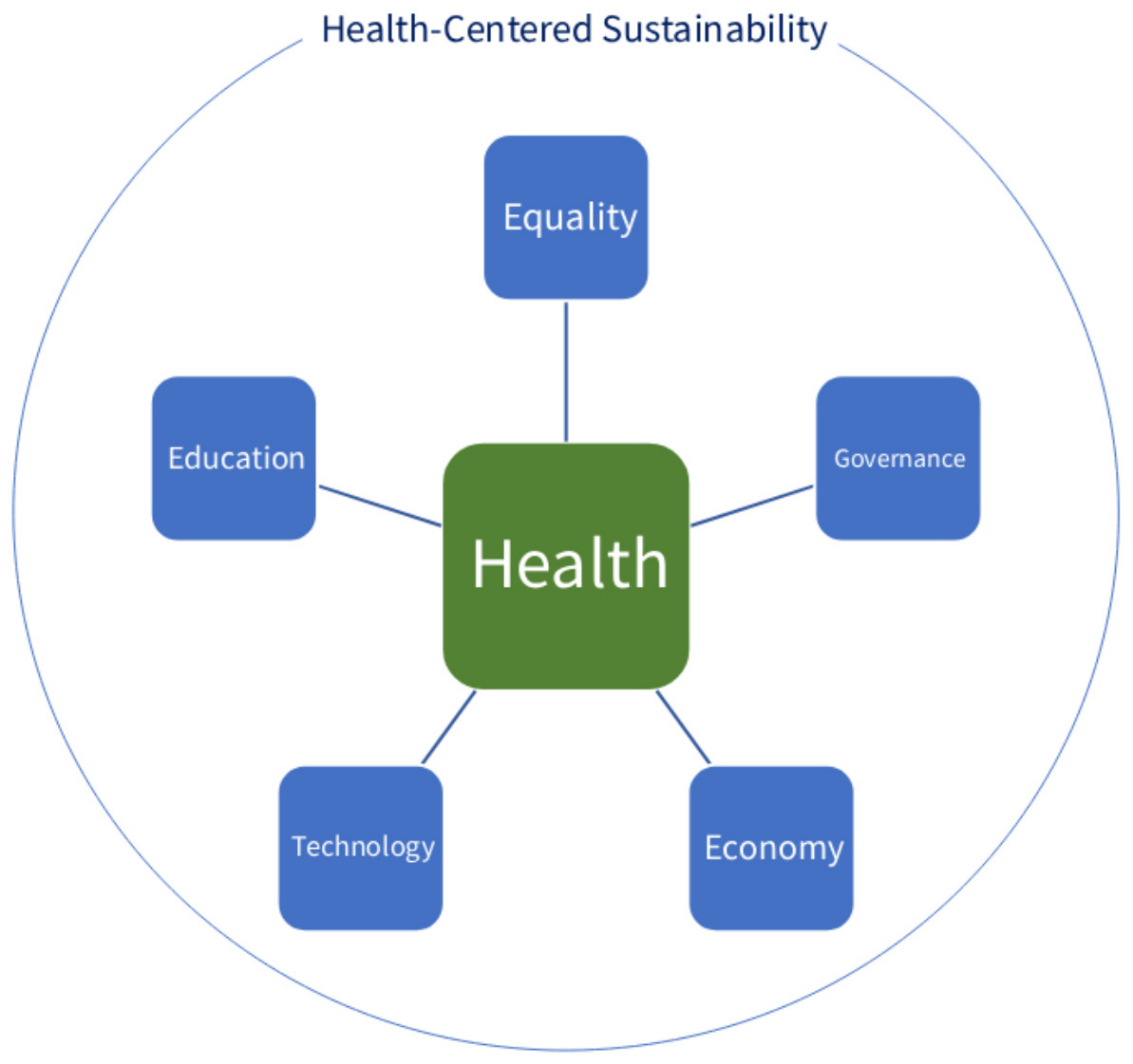
Pillars of health-centered sustainability.

Health and equality are among the most crucial factors of sustainable development. According to the World Health Organization, good health and wellbeing play the most central role in sustainable development, establishing the health-centered sustainability (1). This highlights the synergies flowing from the United Nations' Sustainable Development Goal 3 (SDG 3: Good Health and Wellbeing) toward other elements of sustainable development. These synergistic spillovers support the notions of integrated sustainability perspective and health-centered sustainability perspective (2). Figure 1 presents a health-centered perspective on sustainability, with health at its core. As shown in Figure 1, health, as the core of sustainability, is closely connected to equality, governance, the economy, technology, and education. The connection between health and each of these pillars is explored in this Research Topic, represented as follows.

Equality establishes a pillar of sustainable development, underscoring SDG 10: Reduced Inequality. Beyond the contribution of each SDG 3 and SDG 10, their combination fundamentally determines the level of sustainability through various mechanisms, such as equal distribution of healthcare services. However, evidence indicates that the health sector still suffers from unequal access to medical services, quality of healthcare, and health outcomes, which are substantially influenced by discrimination in socioeconomic, institutional, and regional indicators. The studies in this Research Topic show that imbalanced access to health services is rooted in economic infrastructure, good governance, technology, regional distribution, and social status, rather than in healthcare resources.

Socioeconomic factors have a significant impact on the level of health-sector equality. Access to health services depends substantially on income, education, age, and regional socioeconomic level (Zhang et al.). For instance, groups with lower incomes and less developed regions are more vulnerable to health issues (3). According to this finding, health crises not only underscore the infrastructural gaps but also widen them (Choi et al.). In this way, the disproportionate distribution of healthcare services reflects an imbalance in the allocation of socioeconomic resources. Thus, reducing inequalities in the health sector requires intersectoral interventions in socioeconomic strategies rather than policy-making within the health sector.

Good governance is another factor determining the health outcomes and efficiency (4). From this perspective, the efficiency of health expenditure depends mainly on institutional quality, corruption, and the governance system, thereby rejecting the claim that increased expenditure necessarily improves health outcomes (Köroglu and Bayar). For example, if institutes are highly corrupt, further investment and expenditure in the health sector can not only improve health outcomes but also intensify inequalities in the sector (Yusufu et al.). Therefore, institutional infrastructure is a fundamental element of sustainable development, especially in the health sector, underscoring the roles of good governance, transparency, and accountability.

Another determinant of equality in health outcomes is justice in the regional distribution and allocation of health resources. The health sector suffers from significant discrimination in the distribution of human resources, medical equipment, and healthcare infrastructure, despite widespread coverage of health services. This shows that technical efficiency is not always associated with distributional justice, necessitating the analysis of equality indicators such as the Gini coefficient, spatial accessibility, and efficiency methods (Ba and Luo). Therefore, access to services, especially in less developed regions, is an important prerequisite for sustainable development in the health sector.

In addition to geographical discrimination, the health sector also shows inequalities among people with different levels of vulnerability and demographic characteristics (5). For example, malnutrition and inadequate health conditions for mothers not only affect health outcomes but also perpetuate health inequalities in future generations (Prasetyo et al.). This indicates that equal provision of healthcare for children and their mothers is not a social cost, but a long-term investment in sustainable development.

Technology and education contribute to another aspect of sustainable development in the health sector, though they may increase inequality by widening the digital divide (Zheng et al.). Population health depends significantly on information technology and the digital economy through increased access to information, more efficient healthcare equipment, and reduced health service costs (6). In addition, education level and public knowledge on health considerably improve health status. However, the discriminatory distribution of digitalization and heterogeneous demographic characteristics increases the inequalities in the provision of health services (Yang et al.). Therefore, technological advancement should be effectively combined with equal access to the technologies to enhance the health sector within the sustainable development framework.

In conclusion, sustainable development relies fundamentally on health and equality, especially through socioeconomic development, good governance, reduced digital divide, equitable regional distribution of medical services, and proportionate allocation of healthcare resources among groups with diverse demographic characteristics and vulnerability levels. In the context of sustainable development, the health sector should be strengthened alongside improved equality.
